# New insights into the role and mechanism of macrophage migration inhibitory factor in steroid-resistant patients with systemic lupus erythematosus

**DOI:** 10.1186/ar3828

**Published:** 2012-05-02

**Authors:** Fang-Fang Wang, Li-An Zhu, Yu-Qiong Zou, Hui Zheng, Alisa Wilson, Cheng-De Yang, Nan Shen, Daniel J Wallace, Michael H Weisman, Shun-Le Chen, Liang-Jing Lu

**Affiliations:** 1 Department of Rheumatology, Renji Hospital, Shanghai Jiaotong University School of Medicine, 145 Middle Shan Dong Road, Shanghai 200001, China; 2 Department of Geratology, Renji Hospital, Shanghai Jiaotong University School of Medicine, 145 Middle Shan Dong Road, Shanghai 200001, China; 3 Division of Rheumatology, Cedars-Sinai Medical Center, 8700 Beverly Blvd, Los Angeles, CA 90048, USA

## Abstract

**Introduction:**

Glucocorticoid (GC) therapy remains important in improving the prognosis of patients with systemic lupus erythematosus (SLE). However, some patients do not achieve an effective response with GC treatment, creating an obstacle to the remission of SLE. Identification of the underlying mechanisms responsible for steroid resistance can be significant. Macrophage migration inhibitory factor (MIF) arouses our interest because of its reciprocal relationship with GCs. In the present study, we investigated for the first time whether MIF correlated with steroid resistance in SLE and explored potential mechanisms of action.

**Methods:**

Sixty-two patients with SLE (40 steroid sensitive and 22 steroid resistant) and 21 normal controls were recruited. Serum levels of MIF were measured by ELISA. Cytosolic MIF and IκB expression in peripheral blood mononuclear cells (PBMCs) were determined by western blotting. The electrophoretic mobility shift assay was assessed by NF-κB in nuclear aliquots. Gene silencing was applied to reduce expression of MIF in PBMCs in steroid-resistant patients. PBMCs obtained from steroid-sensitive patients were treated with recombinant human MIF of different concentrations.

**Results:**

MIF levels in serum and PBMCs were higher in steroid-resistant patients compared with steroid-sensitive patients and controls. In contrast to the steroid-sensitive group, NF-κB levels were significantly higher and IκB levels lower in steroid-resistant patients. After MIF gene silencing, IκB levels in cells from steroid-resistant patients were increased. In steroid-sensitive patients, a decrease in IκB levels and an increase in NF-κB expression from baseline were detected in PBMCs treated with a higher concentration of recombinant human MIF. Treatment with recombinant human MIF did not regulate expression of IκB and NF-κB in PBMCs from patients treated with an anti-MIF monoclonal antibody.

**Conclusions:**

Our results indicated that MIF may play a role in the formation of steroid resistance in SLE by affecting the NF-κB/IκB signaling cascade. As a regulator of glucocorticoid sensitivity, MIF may be a potential target for steroid sparing.

## Introduction

Systemic lupus erythematosus (SLE) is a prototype systemic autoimmune disease characterized by autoantibody production. Although the outcome for SLE patients has improved in recent years, SLE continues to profoundly affect health status and the likelihood of disability and premature death. How to ameliorate the reduced quality of life and increase the survival rate in these patients remains a major challenge for rheumatologists. Glucocorticoid (GC) therapy plays an important role in the treatment of SLE, but irreversible side effects limit its use at high doses or over the long term while some patients with highly active SLE do not respond to GC therapy. There is thus a need to investigate the underlying mechanisms behind steroid resistance. Macrophage migration inhibitory factor (MIF) arouses our interest because of its unique relationship with GCs.

Although initially described as a product of activated T cells [1], MIF is now known to be produced by a variety of cell types, predominantly by macrophages [2]. Increasing evidence indicates that MIF is a broad-spectrum proinflammatory cytokine that can increase the expression of a number of inflammatory molecules, including TNFα, IL-6, IL-1β, IL-2, IL-8, and IFNγ [3]. In contrast to other proinflammatory cytokines that are generally suppressed by GCs, MIF expression and secretion are induced by low physiological concentrations of GCs [4,5]. Evidence of upregulation of MIF by endogenous GC has been reported in rat adjuvant-induced arthritis [6]. Regulation of serum MIF by exogenous GC in humans has also been demonstrated in patients with SLE, where it has been observed that serum MIF was influenced by the GC dose even after adjusting for disease activity variables [7]. Despite being induced by GC, MIF exhibits GC-antagonistic effects *in vitro *and *in vivo*. In murine antigen-induced arthritis, GC inhibition of histological severity of disease is reversed by exogenous MIF [8]. Consistent with this finding, Leech and colleagues reported that increased joint inflammation and lethality can be overridden by the neutralization of MIF in the absence of GC in rat adjuvant-induced arthritis [9]. The reciprocal relationship between MIF and GCs in the control of the inflammatory response was also reported in human subjects [10,11]. Several studies to date have revealed that deficiency of MIF, either through genetic deletion or by the anti-sense oligonucleotide, leads to a left-shift in the dose response to GC of macrophage TNF production, which represents the fact that MIF does indeed directly regulate GC sensitivity [6,12].

The mechanism by which MIF counter-regulates the anti-inflammatory actions of GC has not been fully elucidated. However, there are several pathways through which MIF and GCs may interact with each other. One such pathway involves the activation of the transcriptional factor NF-κB. In its inactive state, NF-κB is sequestered in the cytoplasm by the inhibitory protein IκB, and the phosphorylation of the latter results in its ubiquination and degradation by the proteasome, unmasking a nuclear localization signal on NF-κB. In the nucleus, NF-κB binds to DNA sequences called NF-κB elements and is responsible for the transcription of cytokines, chemokines and cell adhesion molecules [13]. The IκB protein binds to activated NF-κB in the cell nucleus, causing dissociation of the latter from the IκB binding sites of the target genes and its subsequent relocation in the cytoplasm [14,15]. GCs prevent NF-κB activation by increasing the expression of IκBα [16,17], which keeps the NF-κB/IκB complex in cytosol and thus prevents the synthesis of inflammatory mediators.

In the present study, we investigated for the first time whether MIF correlated with steroid resistance in SLE and explored the role of MIF and the NF-κB/IκB signaling cascade.

## Materials and methods

### Patients and controls

American College of Rheumatology criteria for SLE and Systemic Lupus Erythematosus Disease Activity Index (SLEDAI) scores were adopted for diagnosis and disease activity assessment of SLE patients, respectively [18,19]. Patients whose SLEDAI score did not decrease after treatment for 1 month with prednisolone (1 mg/kg/day) were defined as steroid resistant (SR), while patients whose SLEDAI score decreased with treatment were defined as steroid sensitive (SS) [20]. A total of 22 patients (18 women and four men) were recruited into the SR group. A total of 40 patients (33 women and seven men) matched for age and gender were selected into the SS group. All 62 patients were seen by the Department of Rheumatology at Renji Hospital. Twenty-seven healthy volunteers, with no signs of acute or chronic disease, served as age and gender controls.

Serum samples and peripheral blood mononuclear cells (PBMCs) were collected at the onset of treatment. The following demographic and clinical data were collected: gender, age, number of years since diagnosis, SLEDAI score before and after steroid treatment, and cumulative dose of prednisolone. Anticoagulated blood samples were collected from patients and controls.

All patients and controls were informed about the purpose of our study and consented to participate in the study. This study was approved by the institutional review board of Shanghai Jiaotong University.

### Isolation and incubation of peripheral blood mononuclear cells

PBMCs were isolated from anticoagulated blood using Ficoll-HyPaque gradient centrifugation (Sigma-Aldrich, St Louis, MO, USA), which was performed at 400 × *g *for 30 minutes at 25°C. The PBMC-enriched interphase was collected and washed with PBS. Trypan blue staining detected the viability of freshly isolated cells. For *in vitro *experiments, PBMCs were resuspended in RPMI 1640 medium (Gibco BRL, Gaithersburg, MD, USA) supplemented with 2 mM L,L-glutamine, 100 μg/ml penicillin G and 100 μg/ml streptomycin, and 100 mM Hepes, 50 μM 2-mercaptoethanol, at a concentration of 5 × 10^6 ^cells/ml. Either recombinant MIF or GC was added to the cells and incubated at 37°C in a 5% carbon dioxide atmosphere.

### ELISA for macrophage migration inhibitory factor

The DuoSet ELISA kit for human MIF (R&D Systems, Minneapolis, MN, USA) was used to measure MIF in serum and cell culture supernates. A 96-well microplate was coated with capture antibody (mouse anti-human MIF) and incubated overnight at room temperature. After a total of three washes with wash buffer, the plate was blocked with reagent diluent and then incubated at room temperature for 2 hours for sample addition. Diluted samples and standards (recombinant human MIF) were added in duplicate to each well for 2 hours at room temperature, followed by the addition of detection antibody (goat anti-human MIF) for another 2 hours. Streptavidin-horseradish peroxidase was then added to each well for 20 minutes at room temperature; the plate was not placed in direct light. Color was developed by incubating with substrate solution for 20 minutes at room temperature. The reaction was terminated with a stop solution, and a microplate reader set to 450 nm (Bio-Rad Laboratories, Hercules, CA, USA) was used to immediately determine the optical density of each well immediately.

### Preparation of cytosolic and nuclear extracts

PBMCs were collected in a 1.5 ml centrifugal tube and washed with 1 ml ice-cold PBS. The suspensions were centrifuged at 750 × *g *for 8 minutes, and pellets were resuspended in 100 μl ice-cold buffer A (10 mM HEPES, pH 7.9; 10 mM KCl; 1.5 mM MgCl_2_; 0.5 mM dithiothreitol) with the addition of phenylmethansulfonyl fluoride. The mixture was then incubated on ice for 10 minutes followed by centrifugation at 18,000 × *g *for 10 minutes. The supernatants (cytosolic extracts. were collected and stored at -80°C. The remaining pellets were resuspended in 20 μl ice-cold buffer B (20 mM HEPES, pH 7.9; 20% v/v glycerol; 420 mM NaCl; 0.5 mM ethylenediaminetetraacetic acid; 1.5 mM MgCl_2_; 0.5 mM dithiothreitol; 0.5 mM phenylmethansulfonyl fluoride and incubated for 30 minutes on ice. After 10 minutes of centrifugation at 18,000 × *g*, the supernatants (nuclear extracts) were collected and stored at -80°C for further study.

### Western blotting

Protein samples (20 μg) were subjected to 15% SDS-PAGE and transferred to polyvinylidene difluoride membranes. The blots were then probed with mouse anti-human MIF (R&D Systems), rabbit anti-human IκB (Santa Cruz Biotechnology Inc., Santa Cruz, CA, USA) or β-actin antibodies, followed by reaction with a horseradish peroxidase-conjugated secondary antibody. Signals were detected using an enhanced chemiluminescence detection kit and were quantified with installed density-analysis software.

### Electrophoretic mobility shift assays

The nuclear extracts (2 μg protein) were incubated with Dig-labeled NF-κB oligonucleotide (Roche, Mannheim, Germany). The assay was performed in a 20 μl total volume containing 2 μg nuclear extract, 4 μl gel shift binding buffer (20 mM Tris-HCl, pH 7.9; 5 mM MgCl_2_; 0.5 mM dithiothreitol; 0.5 mM ethylenediaminetetraacetic acid; 20% glycerol), 1 μg poly(dI-dC), 1 μg poly-L-lysine and 2 μl probe. The reaction was incubated at room temperature for 15 minutes, loaded on a 4% native polyacrylamide gel, and run in a 0.25 × Tris-Borate-EDTA (TBE) buffer. The gel was dried and subjected to autoradiography. NF-κB-specific bands were confirmed by competition with a 50-fold excess of an unlabeled NF-κB probe, which resulted in no shifted band, or by preparing the reaction with excess labeled nonspecific probe, which did not reduce the intensity of the NF-κB band. Quantity One 4.4 software (Bio-Rad Laboratories, Hercules, CA, USA) was used to analyze the results.

### siRNA and plasmid transfection

Three different siRNAs (s8780, s194615, s194614) with putative high silencing efficiency designed for the MIF gene were used (Applied Biosystems, Foster City, CA, USA). We chose to use electroporation, which is the most effective way to introduce siRNA into primary cells. Cells transfected with nontarget double-stranded siRNA served as negative controls. Expression of MIF mRNA was determined by real-time RT-PCR.

### Real-time RT-PCR

Total RNA from PBMCs was isolated with the TRIZOL reagent (Invitrogen, Carlsbad, CA, USA) according to product recommendations. RNA was then reverse transcribed using the Reverse Transcription Kit (Takara Bio Inc., Otsu, Japan). Expression levels of MIF genes were determined by real-time PCR with the SYBR Green PCR Master Mix (Takara Bio Inc.). PCR reactions were carried out in triplicate with the 7900 Real-time PCR System (Applied Biosystems). Forward primer: 5'-GAACCGCTCCTACAGCAAGCT-3'; Reverse primer: 5'-GCGAAGGTGGAGTTGTTCCA-3'. The thermal cycling conditions included 10 min at 95°C and then 40 cycles of amplification for 3 second at 95°C and 20 second at 60°C. The quantity of mRNA was calculated by normalizing the Cycle Threshold (CT) value of MIF to the CT of the housekeeping gene GAPDH in the same sample, according to the following formula: The average GAPDH CT was subtracted from the average MIF CT; the result represents the ΔCT. This ΔCT is specific and can be compared with the ΔCT of a calibration sample. The subtraction of control ΔCT from the ΔCT of interfered group is referred as ΔΔCT. The relative quantification of expression of MIF was determined by using 2^-ΔΔCT^.

### Cell proliferation assay

The Cell Counting Kit-8 (Dojindol Laboratories, Kumamoto, Japan) was employed to measure the concentration of dexamethasone that was 50% lethal to PBMCs from healthy donors, rather than from SS or SR patients with SLE *in vitro*. Cells were seeded in 96-well plates and cultured with various concentrations of dexamethasone. Cells in each group were plated in triplicate. The optical density at 450 nm wavelength, which correlates to the number of viable cells, was measured and cell growth curves were then drawn.

### Statistical analysis

Fisher's exact test or the chi-squared test was used to compare clinical parameters between the SS group and the SR group. *P *< 0.05 was considered significant. The levels of serum MIF, intracellular MIF and intracellular IκB did not conform to a normal distribution, so we conducted Kruskal-Wallis H tests to compare the three groups. If *P *< 0.05, Wilcoxon signed-ranked tests were used to analyze any two groups. Given the multiple groups (SS group, SR group, controls), Bonferroni correction was conducted and *P *< 0.166 was considered significant. Data are expressed as the median (25% to 75% percentiles). SPSS version 10.0 was used for the analysis (SPSS Inc., Chicago, IL, USA).

## Results

### Demographic and clinical characteristics of the SLE patients

Except for a decrease in SLEDAI scores in the SS group (*P *< 0.05) after steroid treatment, there were no significant differences observed between the SS and SR groups (Table 1).

**Table 1:** Demographic and clinical characteristics of the SLE patients

	SS group (*n *= 40)	SR group (*n *= 22)	*P *value
Sex (male/female)	7/33	4/18	0.83
Age (years)	30.78 ± 11.76	29.45 ± 13.15	0.69
Years since diagnosis	3.09 ± 4.77	2.74 ± 4.14	0.77
SLEDAI before treatment	11.88 ± 4.49	12.91 ± 5.37	0.42
SLEDAI after treatment	7.5 ± 3.92	15.05 ± 5.27	0.00
Cumulative dose of prednisolone (mg)	7,719.78 ± 8,731.89	6,372.41 ± 8,925.94	0.84

Data presented as mean ± standard deviation. SLEDAI, Systemic Lupus Erythematosus Disease Activity Index; SR, steroid resistant; SS, steroid sensitive. *P *values calculated using Fisher's exact test or the chi-squared test. *P *< 0.05 was considered significant.

### Serum MIF, cytosolic MIF, cytosolic IκB and nuclear NF-κB expression in patients and controls

**Figure 1:**
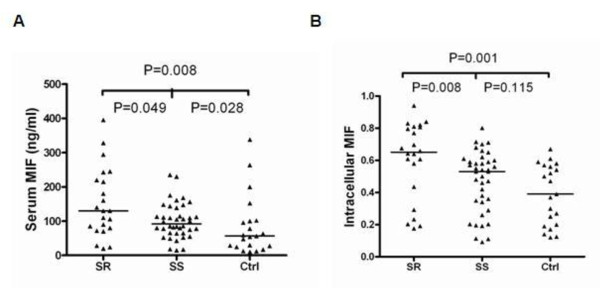
**Serum and cytosolic macrophage migration inhibitory factor levels in peripheral blood mononuclear cells**. **(A) **Serum macrophage migration inhibitory factor (MIF) levels determined by ELISA. **(B) **Intracellular MIF levels determined by western blotting and quantified with installed density-analysis software. The value for each individual is represented as a single point. Horizontal lines represent the median values for each group. Wilcoxon signed-ranks test used to analyze any two groups. Ctrl, healthy donors; SR, steroid-resistant group; SS, steroid-sensitive group.

**Figure 2:**
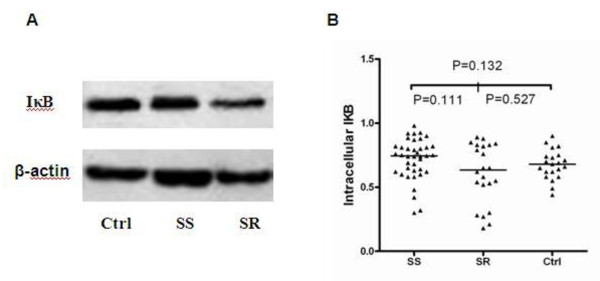
**Cytosolic IκB protein expression in peripheral blood mononuclear cells**. **(A)** Western blotting and **(B) **quantified analysis of the cytosolic IκB protein levels. β-actin was used as the internal control. The value for each individual is represented as a single point. Horizontal lines represent the median values for each group. Wilcoxon signed-ranks test used to analyze any two groups. Ctrl, healthy donors; SR, steroid-resistant group; SS, steroid-sensitive group.

**Figure 3:**
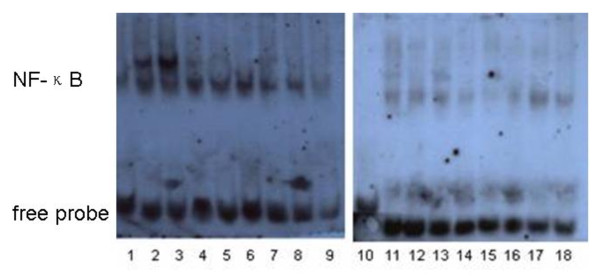
**Electrophoretic mobility shift assay analysis of nuclear NF-κB protein from peripheral blood mononuclear cells**. Lanes 1 and 10, controls; lanes 2 to 8, 17 and 18, steroid-resistant systemic lupus erythematosus (SLE) patients; lanes 9 and 11 to 16, steroid-sensitive SLE patients. Nuclear NF-κB protein levels were quantitated by density-analysis software.

Serum MIF levels in SR SLE patients were 130 (78 to 225.75) ng/ml, higher than the MIF levels in SS SLE patients (91.8 (65.12 to 132.75) ng/ml; *P *= 0.049) and in healthy controls (56.54 (26 to 101) ng/ml; *P *= 0.008). The difference between the SR group and controls was statistically significant (Figure 1A). Likewise, western blotting results showed that cytosolic MIF levels in PBMCs were also significantly higher in SR SLE patients than in SS patients (*P *= 0.008) and controls (*P *= 0.001) (Figure 1B). There was no obvious difference in cytosolic MIF levels in the SS group compared with controls (*P *= 0.115). With regard to the two key molecules in the NF-κB/IκB signal transduction pathway, cytosolic IκB levels in PBMCs appeared to decrease in the SR group relative to the SS group, even though it did not reveal statistically significant differences (*P *= 0.111; Figure 2B). Electrophoretic mobility shift assays results revealed that nuclear NF-κB levels were higher in the SR group than in the SS group (Figure 3).

### Cytosolic MIF and IκB expression after MIF gene-silencing in PBMCs from steroid-resistant patients

**Figure 4:**
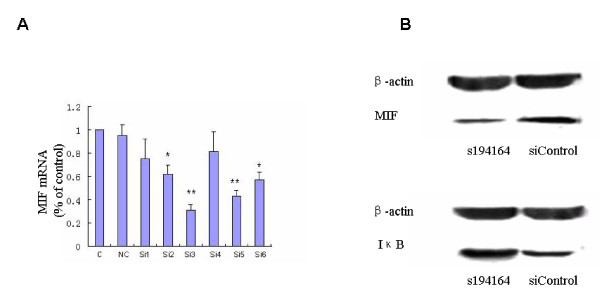
**Cytosolic macrophage migration inhibitory factor and IκB expression after siRNA-mediated gene silencing in steroid-resistant patients**. **(A)** Real-time PCR analysis of macrophage migration inhibitory factor (MIF) mRNA expression in peripheral blood mononuclear cells (PBMCs) from the steroid-resistant (SR) systemic lupus erythematosus group transfected with three siRNAs at different concentrations. C, control; NC, negative control; Si1, 100 nmol/l s8780; Si2, 100 nmol/l s194615; Si3, 100 nmol/l s194614; Si4, 200 nmol/l s8780; Si5, 200 nmol/l s194615; Si6, 200 nmol/l s194614. *P < 0.05, **P < 0.01. s194614 at 100 nmol/l (Si3) had the best silencing efficiency. **(B) **Western blot analysis of cytosolic MIF and IκB levels in PBMCs from the SR group after s194614-mediated MIF gene silencing compared with PBMCs transfected with siRNA controls (siControl).

For initial comparison of the silencing efficiency of siRNAs directed against the MIF gene, three siRNAs against the mRNA encoding MIF were designed and analyzed. PBMCs from several SR patients were co-transfected with three siRNAs respectively at concentrations of 100 and 200 nmol/l. To select the most efficient siRNA we determined silencing efficiency by measuring mRNA expression in infected cells using a target-specific quantitative RT-PCR. As shown in Figure 4A, all three siRNAs were able to downregulate the target gene but s194614 at 100 nmol/l had the best silencing efficiency, so we chose to use this s194614 siRNA. The concentration of dexamethasone that was 50% lethal to PBMCs from healthy donors was an appropriate concentration for observing the impacts of MIF on NF-κB/IκB signaling cascade (that is, the shift in the anti-inflammatory actions of dexamethalone). After siRNA-mediated silencing of the MIF gene and incubation with dexamethasone at 0.42 mg/ml, which was the value for the concentration of dexamethasone that was 50% lethal to PBMCs from healthy donors (cell growth inhibition curve not shown), MIF expression was significantly decreased in SR patients accompanied by an increased cytosolic IκB level relative to controls (data not shown; Figure 4B). Due to large patient variability, we repeated the *in vitro *experiment in multiple donors.

### Cytosolic IκB and nuclear NF-κB expression in PBMCs from steroid-sensitive patients treated with exogenous MIF and anti-MIF mAb

**Figure 5:**
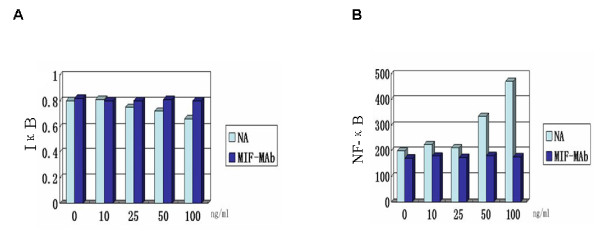
**Cytosolic IκB and nuclear NF-κB protein expression in steroid-sensitive patients after macrophage migration inhibitory factor**. Shift in cytosolic IκB and nuclear NF-κB protein expression in peripheral blood mononuclear cells (PBMCs) from the steroid-sensitive (SS) group after treatment with exogenous migration inhibitory factor (MIF) in the absence or presence of anti-MIF mAb. **(A), (B) **PBMCs from the SS group were first treated with exogenous MIF at different concentrations (0, 10, 25, 50 and 100 ng/ml) for 2 hours with or without previous treatment with anti-MIF mAb and then stimulated with dexamethasone at 0.42 mg/ml for 72 hours. NA, treatment only with exogenous MIF; MIF-MAb, treatment with exogenous MIF and anti-MIF mAb. Cytosolic IκB levels were determined by western blotting and quantified with installed density-analysis software. Electrophoretic mobility shift assay was used to assess the expression of nuclear NF-κB. Quantity One 4.4 software (Santa Cruz Biotechnology Inc., Santa Cruz, CA, USA)analyzed the results.

PBMCs from SS patients were treated with recombinant human MIF at different concentrations (10, 25, 50 and 100 ng/ml) for 2 hours, followed by 0.42 mg/ml dexamethasone for 72 hours. The same treatment was also directed against PBMCs previously treated with anti-MIF mAb. As shown in Figure 5A, B, exogenous MIF attenuated dexamethasone-mediated preservation of cytosolic IκB. The higher concentration of MIF (100 ng/ml) significantly decreased cytosolic IκB, whereas 10 ng/ml had no statistically significant effect (data not shown). There was a reciprocal shift between the amount of NF-κB in the nucleus and the amount of IκB detected in cytosol. In PBMCs previously treated with anti-MIF monoclonal antibody, however, the shift in IκB and NF-κB expression compared with baseline was not significant.

## Discussion

Although GC therapy is the treatment of choice for some autoimmune diseases, some patients show little response to escalating doses of GC therapy. Several mechanisms of steroid resistance have been identified, including genetic susceptibility [21], activation of mitogen-activated protein kinase pathways by certain cytokines [22], increased GC receptor-B expression [23], excessive activation of activator protein-1 [24] and increased *p*-glycoprotein-mediated drug efflux [25,26].

The reciprocal relationship between MIF and GCs in the control of the inflammatory response also makes MIF an interesting factor. In fact, the correlation between increased MIF level, MIF gene polymorphism and steroid resistance has been reported in several autoimmune diseases [10,11,27] and in human CEM T-cell lines [28].

In this study, we investigated whether MIF correlated with steroid resistance in SLE and explored the role of the NF-κB/IκB signaling pathway. Consistent with the results reported by Foote and colleagues [7], we found serum and cytosolic MIF levels were significantly increased in SLE patients. Further analysis showed that MIF expression was higher in SR patients than in SS patients, suggesting that MIF may correlate with steroid resistance in SLE patients. To evaluate the impact MIF exerts on the NF-κB/IκB signaling pathway, the expression of NF-κB and IκB - two key molecules in the signaling cascade - were examined. We detected that cytosolic IκB level was higher in the SS group relative to the SR group while NF-κB expression was lower in the former than in the latter. This finding was consistent with previous reports that GC may induce IκB while inhibiting NF-κB expression [16,17,29,30].

Based on these observations, we interfered with the expression of endogenous MIF. Cytosolic IκB levels in PBMCs were significantly increased in SR patients compared with untreated controls after siRNA-mediated knockdown of MIF gene and treatment with dexamethasone, indicating that to some extent dexamethasone regained its anti-inflammatory actions when accompanied by the downregulation of MIF. Using these results, we can infer that MIF deficiency may upregulate GC sensitivity through interactions with IκB molecules. Aeberli and colleagues, however, reported no direct evidence for the impact of endogenous MIF on transcriptional regulation of IκBα by dexamethasone [12] To further clarify the mechanisms underlying recombinant MIF, different concentrations were added to PBMC cultures from SS SLE patients followed by treatment with dexamethasone. A trend for decreased IκB levels and increased NF-κB expression was seen, suggesting that the anti-inflammatory activities of dexamethasone were weakened in the presence of exogenous MIF. Daun and Cannon reported evidence for MIF reversal of dexamethasone induction of IκBα [31], and Roger and colleagues reported reversal of dexamethasone inhibition of NF-κB by recombinant MIF [6]. Our results are consistent with these previous reports. Amin and colleagues also revealed direct effects of human recombinant MIF on NF-κB pathway molecules [32].

In summary, a comparison of serum and cytosolic MIF expression between SS and SR SLE patients revealed that MIF may correlate with steroid resistance in SLE. Through the downregulation of endogenous MIF in SR patients and the intervention of exogenous MIF in SS patients, we detected the shift in the expression of NF-κB and IκB proteins that played important roles in the anti-inflammatory actions of GCs. The findings suggested that MIF may regulate GC sensitivity in SLE patients via interactions with the NF-κB/IκB signaling pathway. The MIF gene is reported to exert dual impacts on the development and severity of human SLE [33]. Determining the genotype of SR patients and SS patients and exploring the role of MIF as a biomarker for steroid resistance in SLE is therefore promising. As a broad-spectrum proinflammatory cytokine important in innate and adaptive immune responses, the unique relationship with GC implicates MIF as a potential target molecule in patients who show steroid resistance during the treatment of SLE. Leng and colleagues reported that a small-molecule MIF antagonist protects against glomerulonephritis in lupus-prone NZB/NZW F1 and MRL/lpr mice [34]. Development of MIF antagonists for clinical applications in steroid sparing should therefore be pursued. If MIF antagonists can be developed for steroid sparing, the effects of GC may be maximized when treating SR patients with active SLE, potentially improving their prognosis.

## Conclusion

MIF may play a role in the mechanism of steroid resistance in SLE by affecting the NF-κB/IκB signaling cascade. MIF may be a candidate for target therapy in SLE patients who show steroid resistance.

## Abbreviations

ELISA: enzyme-linked immunosorbent assay; GC: glucocorticoid; IFN: interferon; IL: interleukin; mAb: monoclonal antibody; MIF: macrophage migration inhibitory factor; NF: nuclear factor; PBMC: peripheral blood mononuclear cell; PBS: phosphate-buffered saline; PCR: polymerase chain reaction; RT: reverse transcriptase; siRNA: small interfering RNA; SLE: systemic lupus erythematosus; SLEDAI: Systemic Lupus Erythematosus Disease Activity Index; SR: steroid resistant; SS: steroid sensitive; TNF: tumor necrosis factor.

## Competing interests

The authors declare that they have no competing interests.

## Authors' contributions

All authors were involved in drafting the article. L-JL had full access to all of the data in the study and takes responsibility for the integrity of the data and the accuracy of the data analysis. F-FW, L-AZ, Y-QZ and HZ were responsible for the collection of data. The analysis and interpretation of all data were finished by F-FW, L-AZ, AW, C-DY, NS, DJW, MHW, S-LC and L-JL. All authors read and approved the final manuscript.
